# Private Video Consultation Services and the Future of Primary Care

**DOI:** 10.2196/19415

**Published:** 2020-10-01

**Authors:** Chris Salisbury, Anna Quigley, Nick Hex, Camille Aznar

**Affiliations:** 1 Centre for Academic Primary Care, Department of Population Health Sciences Bristol Medical School University of Bristol Bristol United Kingdom; 2 Ipsos MORI Social Research Institute London United Kingdom; 3 York Health Economics Consortium York United Kingdom

**Keywords:** remote consultation, primary health care, general practice, delivery of health care, access to health care, mobile phone

## Abstract

In many countries, private companies provide primary care services based predominantly on offering video consultations via smartphones. One example is Babylon GP at Hand (BGPaH), which offers video consultations to National Health Service patients, 24 hours a day, and has grown rapidly in London over the last 3 years. The development of this type of service has been controversial, particularly in the United Kingdom, but there has been little formal published evaluation of these services in any country. This paper outlines the main controversies about the use of privately provided video consultation services for primary care and shows how they are informed by the limited evaluations that have been conducted, particularly the evaluation of BGPaH. This paper describes the advantages of these services in terms of convenience, speed of access, the ability to consult without traveling or face-to-face patient-doctor contact, and the possibility of recruiting doctors who cannot work in conventional settings or do not live near the patients. It also highlights the concerns and uncertainties about quality and safety, demand, fragmentation of care, impact on other health services, efficiency, and equity. There are questions about whether private primary care services based on video consultations have a sustainable business model and whether they will undermine other health care providers. During the recent COVID-19 pandemic, the use of video consulting has become more widespread within conventional primary care services, and this is likely to have lasting consequences for the future delivery of primary care. It is important to understand the extent to which lessons from the evaluation of BGPaH and other private services based on a *video-first* model are relevant to the use of video consulting within conventional general practices, and to consider the advantages and disadvantages of these developments, before video consultation–based services in primary care become more widely established.

## Primary Care Based on Video Consultations

In the United Kingdom, recent policy has strongly promoted *digital-first* health care under the National Health Service (NHS), arguing that this will improve access and convenience for patients while also increasing efficiency [1-3]. Private companies such as Livi and Pushdoctor have contracted with groups of general practices to offer video consultations free to patients under the NHS. In these models, video consultations are provided by doctors working anywhere in the country, in addition to conventional care provided by patients’ usual doctors. The best-known example in the United Kingdom is Babylon GP at Hand (BGPaH), which provides almost all services on behalf of a general practice in London, under a digital-first model whereby almost all consultations are initially conducted using a smartphone [4]. Key features of BGPaH are shown in Textbox 1. Driven by a strong advertising campaign, this service has grown very rapidly in London since 2017 and has recently expanded to Birmingham.

**Summary of Babylon GP at Hand. GP: general practitioner.**

Key features of Babylon GP at Hand:

- Provides a web-based symptom checker.
- Offers video or telephone consultations with a General Practitioner (GP) 24 hours a day, usually within 2 hours of making a request.
- Babylon GP at Hand (BGPaH) GPs make referrals and provide prescriptions in the same way as conventional GPs, sending them electronically.
- When necessary (usually only after a video or telephone consultation), patients can have a face-to-face consultation at one of 7 locations across London or one in Birmingham.
- Patients have to deregister from their previous National Health Service (NHS) general practice when they register with BGPaH.
- Under the NHS GP Choice policy, patients can register with BGPaH wherever they live, providing they can travel to one of the clinic sites within 40 min.
- If patients need urgent help and are not able to visit a clinic, or need a home visit and do not live near a BGPaH clinic, they have to contact the NHS 111 national telephone helpline to arrange help from other NHS providers.
- Patients who need frequent face-to-face consultations or have difficulty traveling to a clinic are encouraged by BGPaH to register elsewhere.
- Patients requiring a face-to-face consultation for specific purposes (eg, a cervical smear) contact a support team to make an appointment at one of the five clinics.
- Consultations are provided by over a hundred GPs, mostly working from home from anywhere in the country. Some GPs work at a central hub in London, which also manages test results and repeat prescriptions.

Similar developments have occurred in many other countries, with private companies offering video consultations via smartphones instead of conventional face-to-face appointments for primary care. Examples include Dr on Demand in the United States; Curon in Japan; Ping An Good Doctor in China; and KRY, which operates in several European countries. Often backed by venture capital, these companies are expanding rapidly into new regions. The aim of this paper is to consider the implications of these developments for the future of primary care. It particularly focuses on the United Kingdom, and lessons learnt from the evaluation of the BGPaH service in London, but also refers to relevant findings from evaluations of similar services in other countries.

## Controversies

The growth of private companies offering video consultations for primary care has been controversial. Concerns have been expressed about the safety and effectiveness of managing patients by telephone and video, and there have also been concerns that services focused on speed of access will undermine continuity of care [5,6]. New commercial services could compete for staff at a time when shortages of doctors threaten the viability of some general practices. An underlying concern in the United Kingdom is that the involvement of commercial companies offering video consultations undermines the public service ethos of the NHS and increases fragmentation of services.

However, others have argued that video consulting represents the future of health care [7,8] and that criticisms are based on resistance to change, with health services having been slow to exploit the potential of technology to improve efficiency and convenience for consumers [7,9].

Some of these arguments have conflated 3 different issues. First, there is the issue of private companies providing a new model of care, either in competition with conventional services or as an adjunct to conventional care. Second, there is debate about the appropriate role of video consultations in primary care. The third issue is about *digital-first* access models in which patients are expected to have an initial contact by video, telephone, or asynchronous web-based message before being offered a face-to-face consultation only when necessary.

These 3 issues are related because the new model provided by services such as BGPaH depends on video consultations and digital-first approaches. However, the recent COVID-19 pandemic has greatly accelerated the use of remote consultations and digital-first approaches by conventional general practices. In the past, the introduction of these approaches has been slow and patchy, but during the pandemic, doctors in many different countries have rapidly turned to telephone and video consultations because of the need to manage large numbers of patients without face-to-face contact [10]. Video consultations may be offered as an option or within a digital-first model. In this paper, we focus on private companies offering primary care on a *video-first* model, particularly lessons learnt from the evaluation of BGPaH. However, some of the points discussed will also be relevant to the provision of video consultations within a conventional primary care model.

## Evaluation

Despite the rapid growth of companies offering primary care video consultation services and the controversy surrounding them, there has been almost no rigorous evaluation of these services in any of the countries where they have been established. Some of the most detailed evidence comes from an independent evaluation of BGPaH, which was commissioned by the NHS and published on the website of a local clinical commissioning group in May 2019 [11]. Textbox 2 summarizes the key components of this evaluation, which was designed to understand the impact of BGPaH on patients, the general practitioner (GP) workforce, and the wider health care system. The evaluation report and an annex providing details of the full results are available elsewhere [11,12].

**Components of Babylon GP at Hand evaluation. GP: general practitioner.**

- *Patient experience survey*: Web-based survey of 1452 Babylon GP at Hand (BGPaH) patients (1452/23,073, 6.29% response rate) to quantitatively assess their experience, compared with a similar patient cohort responding to the National Health Service (NHS) GP Patient Survey [13], matched using propensity score matching.
- *Qualitative interviews*: In-depth interviews with 12 general practitioners, a nurse and a member of operational staff from BGPaH, 32 current patients (including 3 in the process of deregistering), and 4 patients who had deregistered from BGPaH, along with site visits to the BGPaH hub and one clinic.
- *Analysis of secondary data*: Analysis of routine data sets about activity and synthesis of NHS England analytical work using nationally held data.

There were significant limitations to the BGPaH evaluation due to the availability of data and the short timescale of the evaluation. The patient survey had a very low response rate, which raises concerns about the representativeness of the views expressed. It was not possible to make comparisons between responders and nonresponders, but survey participants were matched using propensity score matching to patients with similar characteristics receiving conventional care. Babylon was only able to provide limited data about patients’ face-to-face consultations, and no information was available about patients’ presenting problems, health outcomes, or referrals to other NHS services.

Despite these limitations, the BGPaH evaluation provides useful information as a case study to inform current debates about the implementation of video-first services in primary care. In the United States, there have been brief reports about digital primary care based on video consultations from organizations such as Kaiser Permanante [14-16], Jefferson Health [17,18], and the Veteran’s Administration [19]. Several digital health platforms have been established in Sweden, and these have been reported in a descriptive evaluation, but this does not distinguish between video, audio, and text-based web-based consultations [20]. None of these reports represent a comprehensive evaluation, and all have significant limitations, but they do provide insights into some of the controversies.

## Advantages of Video Consultation Services in Primary Care

A primary care service based mainly on a digital-first model (including video and/or telephone consultations) has obvious advantages. It provides patients with convenient access to health care advice without leaving home or work, offering savings in time and travel costs to patients and productivity gains for society [21]. It improves access in sparsely populated areas where physically getting to a GP may be problematic. As clinicians can work from anywhere, this makes it possible to recruit doctors who cannot work in conventional settings and to provide care in areas where it is difficult to recruit sufficient GPs. In the recent COVID-19 pandemic, remote consultations by video and telephone made it possible to avoid direct patient-doctor contact.

## Patient Experience

Patients choosing to use private video consultation services appear to be generally very satisfied with their care, although it is important to remember the limitations of the evidence (particularly nonresponse bias). Table 1 shows responses from participants in the survey of users of BGPaH [11] compared with matched patients responding to the national GP Patient Survey [13]. Overall, 71.49% (1038/1452) of BGPaH survey respondents described the quality of care they received at BGPaH as being better than that they received at their previous general practice, whereas only 10.67% (155/1452) described it as worse. This greater satisfaction appeared to be driven by the speed and convenience of the service, the quality of interpersonal care, and the length of consultations. These advantages were not specific to video consultations, with many patients choosing telephone consultations with BGPaH.

**Table 1:** Patients’ experience of Babylon GP at Hand compared with matched respondents from the national GP Patient Survey (summary data). GP: general practitioner.

Question	Response option	GPPS^a^ respondents^b^		BGPaH^c^ respondents	
		n (%)	N	n (%)	N
Overall, how would you describe your experience of making an appointment?	Very good	258 (23.39)	1103	717 (65.48)	1095
Overall experience	Very good	402 (34.69)	1159	682 (58.19)	1172
Thinking about the reason for your last general practice appointment, were your needs met?	Yes, definitely	622 (55.83)	1114	663 (63.69)	1041
At your last appointment, how good was the doctor at giving you enough time^d^	Very good	361 (40.7)	887	546 (61.8)	884
At your last appointment, how good was the doctor at listening to you^d^	Very good	395 (44.6)	885	571 (64.6)	884
At your last appointment, how good was the doctor at treating you with care and concern^d^	Very good	385 (43.7)	882	553 (62.8)	880
During your last general practice appointment, did you have confidence and trust in the health care professional you saw or spoke to?^d^	Yes, definitely	507 (57.2)	886	638 (71.9)	887

^a^GPPS: General Practitioner Patient Survey.

^b^Propensity matched sample of patients from the GP Patient Survey [13] who were resident in London and had similar characteristics to respondents in the BGPaH survey. Matching variables used data available in both surveys, including age, gender, ethnicity, religion, sexuality, work status, whether there were children in the household, whether the respondent was a carer, and whether the respondent had a limiting long-term illness.

^c^BGPaH: Babylon GP at Hand.

^d^Responses from patients who had a consultation with a general practitioner.

Less positive views were expressed in interviews by some patients who needed face-to-face consultations or had complex needs (Textbox 3). Although some patients were concerned about a lack of continuity of care, most had actively chosen speed of access over continuity. Some patients experienced problems with integration between the digital and face-to-face services, particularly issues such as providing urine samples, following up test results, and arranging hospital referrals.

High levels of patient satisfaction have also been found in evaluations conducted in the United States [16-19], similarly driven by the increased speed and convenience of video consultations compared with waiting for a face-to-face appointment.

**The views of Babylon GP at Hand patients and doctors. GP: general practitioner.**

Patients

- “I can only get doctors’ appointments on weekdays during office hours so I couldn’t register with the doctors. So I thought this was the best idea I’ve ever seen.”
- “I would never have made an effort to see a GP - too much effort to leave work, make appointment and plan around it. Having phone consultations makes things a lot easier - convenience, evening weekends, not having to miss work or having to trek to where GP is.”
- “You click a button and get an appointment in 10 minutes, that’s their selling point, but there's nothing for long-term health management.”
- App experience great for arranging telephone or video consultation but when referred for a face-to-face examination it was a nightmare getting an appointment! No convenient times outside of working hours during the week. Had to wait ages to get something that would work for me.”

General Practitioners

- “I was dissatisfied with my practice that I'd been at. (...) I felt underappreciated. I felt overworked and under-remunerated for the services that I gave in my own time, whereas all of those things were a bit different. The ethos here is very, very different.”
- ”General practice has suffered a lot in the last ten years, and it’s really hard for GPs to do the job they want to do as best as they can. (...) I was doing two years in different salaried jobs and it was really hard and I didn’t feel valued, didn’t feel like I was delivering what I wanted to for patients. Actually, this was the first time as a GP I’ve actually felt like I can do that, I can deliver the care.“

## Impact on Emergency Departments

**Figure 1:**
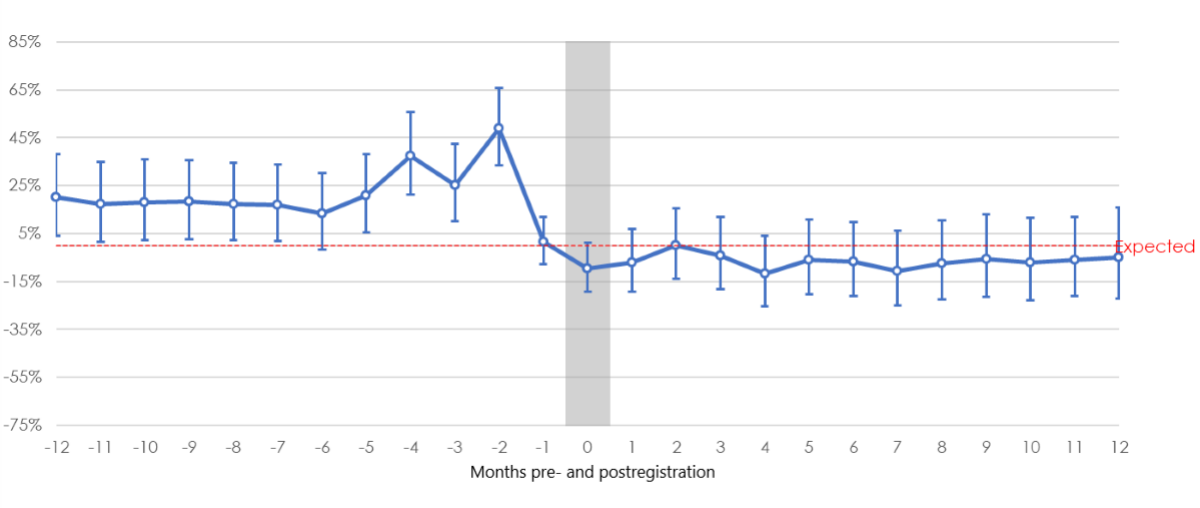
Babylon GP at Hand emergency department attendance rates compared with newly registered London patients (age-sex standardized). Source: National Health Service England. GP: general practitioner.

One potential advantage of video consultation services is that by improving access to general practice, they could reduce the use of emergency departments for primary care problems. However, there is very limited evidence to support this claim. For example, although analysis in the BGPaH study showed an apparent reduction in the use of emergency departments (Figure 1), the evaluators noted that there were a number of potential confounders in the data that meant robust conclusions could not be drawn. It was also notable that BGPaH patients had below average use of hospital admissions and outpatient attendances both before and after registration (age-sex standardized), reflecting that they are a generally healthy population.

## Uptake

One of the most notable features of BGPaH is the speed at which it has attracted patients, when other studies have highlighted very low uptake of video consultations. For example, Kaiser Permanente has offered video consultations since 2014, but between 2015 and 2017, these accounted for less than 1% of all office consultations [15]. In the United Kingdom, uptake of video consultations was very low when offered by conventional general practices before the COVID-19 pandemic [22,23]. In Sweden, the use of digital consultations has been increasing for several years but still represented only 2% of all primary care consultations by 2017 [20]. A consistent finding has been that introduction of video consulting has been impeded by technical problems [23,24]. The apparent success of BGPaH may be because the whole process of care has been redesigned around a new technology-enabled model with a very strong focus on convenience for patients. Experience of technological innovations in other settings shows that these are only likely to improve services when they are used to enable whole-system redesign, rather than being bolted on to existing systems and processes [9]. It is notable how, in the recent pandemic, many of the issues that impeded the introduction of video consultations seem to have been rapidly overcome through a combination of technical innovation, entrepreneurship, an obvious incentive for clinicians to avoid face-to-face contact, and a pragmatic relaxation of regulatory objections.

## Equity

The vast majority (94%) of patients registered with BGPaH are aged under 45 years; two-thirds live in more affluent areas; and 84% are in paid work, and they have substantially fewer long-term health conditions than age-sex matched controls, except for asthma. Currently, only 0.28% of BGPaH patients are aged over 70 years, compared with 12% in an average English general practice [25]. These findings suggest that this type of video-first service is particularly suitable for patients with less complex health care needs but also raise questions about equity of access in relation to need. In Kaiser Permanente, the use of electronic tools is highest among young adults and white patients and lower among older patients and those from African American or Latino ethnic groups [14]. In Sweden, there is a significantly positive relationship between the use of digital care and affluence [20].

## Quality and Safety

A number of authors have questioned the quality and safety of digital consultations [26,27] and argued that these should require the same level of evidence as other medical innovations [26]. Although there are published frameworks for the evaluation of digital health systems, which include the need for evidence about usability, effectiveness, and safety in real-world implementation [27,28], this evidence is largely absent for video consultation systems. A recent Scottish study concluded that video consultations dealt with fewer problems and achieved lower scores on measures of consultation quality than face-to-face consultations and were most suitable for simple problems [23]. Babylon has published their own analysis of the safety of the automated triage software used in their digital health platform [29], but this was not peer-reviewed and the findings have been subject to criticism [27]. However, the evaluation of BGPaH highlights several positive aspects of the service [11], and a recent inspection by the Care Quality Commission rated BGPaH as good overall [30].

## Supply-Induced Demand

There is some evidence that, by reducing the threshold to access care, video consultation services may lead to increased consultation rates through supply-induced demand. In a patient survey, 47% of BGPaH patients reported that they used the service more regularly than their previous practice (8% said they used it less regularly), and interviews with patients also gave examples of increased frequency of use. The estimated mean annual consultation rate of patients with BGPaH was 4.3 consultations per annum, higher than the national average for patients of their age [31]. In Kaiser Permanente, the number of *virtual visits* after offering internet, mobile, and video access routes increased from 4.1 million in 2008 to 10.5 million in 2013, but there was no associated reduction in face-to-face consultations [14]. Similarly, an evaluation of BlueShield in California demonstrated a substantial increase in utilization and health care spending [32]. This increased use of primary service utilization may be an advantage if it addresses unmet need but is problematic if it diverts time and resources away from patient groups with greater needs. This is an issue of cost-effectiveness but cannot be assessed without information about patient outcomes or impacts on other health services.

## Cream-Skimming?

In the United Kingdom, the growth of BGPaH has been particularly controversial because of claims that it threatens the viability of conventional practices by targeting young and healthy patients, leaving conventional practices to see older patients and those with more complex problems who generate most work [33,34]. However, this is arguably a problem with the way in which GPs in the United Kingdom are paid under the NHS, rather than the BGPaH model itself. The current payment formula for general practice in the United Kingdom takes into account the age and sex and the health needs of a patient population at an area level, but it does not effectively adjust for the different health needs of individual patients. The problem of how to fairly reimburse health providers in ways that support the appropriate use of video consultations without increasing health system costs is being debated in the United Kingdom [35,36] and several countries [14,20]. In relation to the argument about cream-skimming, it is notable that BGPaH does not appear to attract patients who generate less work—if anything, their patients have higher consultation rates than average patients of similar age and sex, although they have fewer long-term health conditions.

## Is the Improved Access Due to Video Technology or Greater Investment?

The main aim of primary care services based on a video-first model is to improve access to care. Video (and telephone) consultations make it possible for patients to see a doctor without traveling and losing time from other activities. It is noteworthy that although the BGPaH model defaults to a video consultation, the evaluation found that almost as many patients had used a telephone consultation as had used a video consultation. The main advantage for patients appeared to be the ability to get any sort of consultation quickly, rather than the mode of consultation. It is not obvious why providing consultations by video should reduce delays to speak to a doctor, if this is due to a shortage of GP availability. GPs working for BGPaH see fewer patients in a 4-hour shift than GPs working in most conventional NHS practices. It is possible that the key issue that improves access is the re-engineering of the organizational model and access pathway, rather than the use of technology. Alternatively, it could be that the improved access is due to greater investment in additional capacity and that similar improvements could be made with increased investment in conventional care.

## The Sustainability of New Models of Care

One of the reasons that the growth of BGPaH has been controversial in the United Kingdom has been a concern about a gradually increasing provision of segmented services by private companies and a lack of transparency about their business models. There are assumptions that digital primary care will be more efficient than conventional models of care, but the evidence available so far does not support this hypothesis [37]. No details of the costs and productivity of staff were available for the evaluation of BGPaH because they were considered commercially confidential. Although the per-consultation cost of a video consultation at Kaiser Permanente is less than a face-to-face consultation, increased consultation rates mean that cost savings have not materialized [14]. Many of the new video-based primary care services are provided by start-up companies that are trying to disrupt what they see as entrenched service models. Backed by large investments of cash [38], they are competing to gain market share, and it is not clear whether their current operations are profitable or instead being provided as loss-leaders. In the context of a state-funded service such as the NHS, it is important to understand far more about the costs of delivering smartphone-based services to ensure that they are sustainable, before they become indispensable after having displaced other services.

A further concern has been that private services such as BGPaH could strip the NHS of staff by offering more flexible and convenient working conditions. In the BGPaH evaluation, we observed that BGPaH GPs tended to be younger than the general GP workforce, and more than two-thirds are female. Many were attracted to the job because it offered a good work-life balance. Most were very satisfied with working for Babylon, contrasting the autonomy, training, flexibility, and good working conditions with the long hours and increasing workloads they experienced in conventional general practice. These findings may have important implications for how conventional services need to improve working conditions to retain staff.

## Continuity of Care

Conventionally, the existence of a relationship built over time between a patient and their doctor has been viewed as one of the defining features of general practice. Continuity of care is associated with benefits in patient satisfaction, adherence to medical advice, use of hospital services, and health outcomes [39]. However, continuity of care in conventional general practices in the United Kingdom is declining rapidly [40], and in the evaluation of BGPaH, it was clear that continuity was not a priority for most patients choosing this service. The growth of BGPaH and other private video consultation services is one manifestation of a change in relationships between patients and their general practitioners, with many patients choosing a more impersonal but potentially more convenient service. It is possible that there will be a loss of the benefits associated with continuity of care. Although no such adverse effects were observed in the evaluation of BGPaH, it will be important to monitor these effects over a longer period.

## Implications of Private Video-First Consultation Services for the Future of Primary Care

Evaluation suggests that video-first consultation services for primary care are associated with high levels of satisfaction among the type of patients who prefer this approach, and this is generally a young and healthy population. However, this may not necessarily provide a model for designing services for the majority of patients who use primary care and who may have different needs or priorities. Private video consultation–based services provide some aspects of primary care to only a limited extent and rely on the continued existence of conventional practices to provide these. Smartphone-based services focusing on addressing specific presenting complaints may not provide opportunistic health care, which is a central role of general practice. Older patients with chronic diseases make up the bulk of the general practice workload. These patients are much less likely to choose to use video consultations than younger patients [11], and they also place a higher priority on relational continuity of care from a doctor they know [41].

One possible scenario for the future of primary care is the development of different types of services tailored to the needs of different population groups. However, this also has several potential disadvantages, including the loss of a single point of contact for health care provided by conventional general practice; the potential for individuals to fall into the gaps between different services, each with a defined remit; and the need for patients to transfer between services as their health care needs change. This particularly applies to older patients, but is also relevant to many other patient groups, particularly those who are vulnerable or have complex needs.

Due to the COVID-19 pandemic, many conventional general practices have rapidly implemented similar *digital-first* access pathways and remote consultations, including by video. What lessons can we learn from services such as BGPaH that are relevant to video consulting in conventional general practices? First, it is feasible to use video consultations for many types of consultations, and it is likely that a greater use of these consultations will continue in the future. Second, although the rapid implementation of video consulting during the pandemic has been driven by expediency, it should not be assumed that it necessarily provides optimal or more efficient care. Third, it is notable that even in a service such as BGPaH marketed around the offer of video consultations, many patients prefer the simpler technology of the telephone. Fourth, it is important to distinguish between video consultations as a technology and a service delivery model in which video consultation is the default mode of consulting. If digital consultations are offered as a choice, within a general practice that offers all types of consultation, they should improve access for patients who prefer this option. However, if patients are required to use digital services before accessing a face-to-face consultation (the *digital-first* model as used by BGPaH), this could favor patients with the fewest needs and increase health inequalities.

Private companies providing video consultations for primary care have acted as a challenge to conventional general practice, and the COVID-19 pandemic has further accelerated similar changes in service delivery in these conventional practices. However, there remain questions about important issues such as the quality, safety, and efficiency of video consultations; supply-induced demand; continuity of care; and the appropriateness of video consultations for different types of problems and patients, and these questions require further research. There are also concerns about the implications for the wider health care system of private companies providing video-first services that tend to address the needs of a specific segment of the population. In advance of evidence about these key issues, the growth of private companies providing video-first primary care should be managed cautiously and accompanied by much more extensive and rigorous independent evaluation than has been undertaken so far.

## Abbreviations

BGPaH: Babylon GP at Hand
CCG: Clinical Commissioning Group
GP: general practitioner
NHS: National Health Service
NIHR: National Institute for Health Research
